# Changes in Ras and Ras-Associated GTPases During Maturation of Porcine Cumulus Cells and Oocytes

**DOI:** 10.3390/ani16142125

**Published:** 2026-07-09

**Authors:** Yuna Nam, Hyeonseo Song, Eunju Seok, Sang-Hee Lee

**Affiliations:** 1 College of Animal Life Sciences, Kangwon National University, Chuncheon 24341, Republic of Korea; yn1619@kangwon.ac.kr (Y.N.); marry040320@kangwon.ac.kr (H.S.); yjchsuk@kangwon.ac.kr (E.S.); 2 School of Information and Communication Technology, University of Tasmania, Hobart, TAS 7005, Australia

**Keywords:** cumulus cells, oocytes, Ras, pig, maturation

## Abstract

Porcine cumulus–oocyte complexes (COCs) undergo dynamic morphological and molecular changes during maturation; however, cell type- and stage-dependent transcript patterns of Ras-related factors in cumulus cells and oocytes remain unclear. This study investigated changes in Ras-related factors during porcine in vitro maturation (IVM). Porcine COCs were cultured under IVM conditions supplemented with follicle-stimulating hormone (FSH), human chorionic gonadotropin (hCG), and epidermal growth factor (EGF). Cumulus cells and oocytes were collected at 0, 22, and 44 h of IVM, and cumulus expansion was monitored using time-lapse imaging. Hormone receptor-related factors, *CX43*, Ras family members, and their RasGAP and RasGEF regulators were analyzed using RT-PCR and qPCR with band-intensity analysis. Progressive cumulus expansion was observed during IVM. In cumulus cells, *R-Ras* transcript abundance increased at 22 and 44 h, whereas *RASA1* transcript abundance was lower at 44 h. In oocytes, *R-Ras* and *H-Ras* transcript abundance decreased during maturation. These findings indicate that selected Ras family members and their regulators exhibit distinct cell type- and stage-dependent transcript patterns during porcine COC maturation. These transcript-level findings provide a basis for future protein-level and functional analyses.

## Simple Summary

Porcine oocyte maturation depends on coordinated changes in the oocyte and surrounding cumulus cells, but the signaling molecules involved in these changes are not fully understood. In this study, we investigated whether Ras-related factors change differently in cumulus cells and oocytes during maturation of porcine cumulus–oocyte complexes. We found that Ras-related transcripts showed cell type- and stage-dependent patterns. In cumulus cells, selected Ras-related transcripts changed during maturation, whereas oocytes showed different molecule-specific temporal patterns. In particular, *R-Ras* transcript abundance increased in cumulus cells but decreased in oocytes. These findings provide basic information on the different Ras-related transcript patterns observed in cumulus cells and oocytes during porcine oocyte maturation and may help improve our understanding of reproductive biology in pigs.

## 1. Introduction

Mammalian oocyte maturation is a highly coordinated biological process involving nuclear and cytoplasmic events essential for acquiring developmental competence [1]. In mammals, oocytes normally mature in close association with surrounding cumulus cells, and these two cellular compartments form cumulus–oocyte complexes (COCs) [2]. Within COCs, cumulus cells and oocytes communicate bidirectionally through direct cell–cell contact and paracrine signaling, thereby regulating metabolic interactions, meiotic progression and cytoplasmic maturation [3,4]. Successful oocyte maturation depends on the coordinated responses of cumulus cells and oocytes [2,4]. Therefore, analyzing the signaling networks in each cellular compartment is essential for understanding the molecular basis of oocyte competence.

Small GTPases, including Ras proteins, function as molecular switches that couple extracellular stimuli to downstream effector pathways [5]. In porcine COCs, gonadotropin- and epidermal growth factor (EGF)-related stimulation rapidly alters cumulus cell gene expression and promotes extracellular matrix formation, cumulus expansion, and meiotic progression [6,7]. The contrasting physiological states of cumulus cells and oocytes make Ras-related regulation particularly relevant to COC maturation. Cumulus cells remain transcriptionally responsive to the maturation environment and mediate bidirectional communication with the oocyte through junctional and paracrine mechanisms [8,9]. In contrast, fully grown oocytes are largely transcriptionally quiescent and rely on the selective translation of stored maternal mRNAs during meiotic maturation [10,11]. Because Ras effector pathways regulate extracellular signal-regulated kinase (ERK) signaling and cytoskeletal organization [5], the temporal transcript patterns of Ras-related factors may differ between the somatic and germ-cell compartments as maturation proceeds. However, the cell type- and stage-dependent regulation of Ras isoforms and their Ras GTPase-activating protein (RasGAP) and Ras guanine nucleotide exchange factor (RasGEF) regulators during porcine in vitro maturation (IVM) remains poorly understood, providing a rationale for investigating these factors separately in cumulus cells and oocytes.

The classical Ras family includes H-Ras, K-Ras, and N-Ras. Related Ras subfamily members, such as R-Ras, are involved in the regulation of cellular morphology, adhesion, and signaling specificity [12]. Ras proteins cycle between an inactive guanosine diphosphate (GDP)-bound and active guanosine triphosphate (GTP)-bound states, thereby functioning as on/off molecular switches [5,12]. Evidence linking individual Ras isoforms to oocyte maturation is uneven and highly context-dependent. In Xenopus, activated H-Ras induces meiotic M-phase entry in fully grown oocytes, whereas H-ras (val12) induces cytoplasmic activation-associated events without complete nuclear maturation in smaller, developmentally less competent oocytes, indicating that the response to H-Ras depends on the developmental state of the oocyte [13,14]. Evidence concerning K-Ras is less consistent. A peptide corresponding to the K(B)-Ras hypervariable region inhibited insulin-dependent but not progesterone-dependent maturation, whereas a later study found that a cytosolic GTP-bound Xenopus K-Ras mutant inhibited progesterone-induced and H-RasV12-induced maturation but did not itself induce meiotic resumption [15,16]. These contrasting findings suggest that the apparent contribution of K-Ras depends on the inducing stimulus, the type of experimental interference, and the intracellular targeting of the Ras construct. In contrast, R-Ras has been investigated primarily in somatic cells, where active R-Ras promotes focal-adhesion formation and cell spreading. R-Ras is also preferentially targeted to focal adhesions, unlike activated H-Ras and K-Ras, indicating isoform-specific spatial regulation [17]. In mammalian ovaries, functional studies have focused mainly on Ras/K-Ras in follicular somatic cells, where follicle-stimulating hormone (FSH)-induced Ras activation contributes to granulosa cell differentiation, and dysregulated sustained K-Ras activation disrupts follicular development and ovulation [18,19]. Recent porcine studies have examined Ras-family transcripts in whole COCs or isolated cumulus cells; however, they have not compared the time-dependent transcript patterns of individual Ras isoforms and their regulators in separately analyzed cumulus cells and oocytes [20,21]. This uneven evidence across isoforms, stimuli, species, and cell types provided the rationale for comparatively examining H-Ras, K-Ras, N-Ras, and R-Ras during porcine IVM.

The Xenopus studies described above provide proof-of-principle that Ras can modulate meiotic entry, but they also indicate that the presence of Ras-related machinery alone does not determine meiotic competence. Comparable membrane-associated p21 Ras abundance and GTPase activity in small and fully grown Xenopus oocytes, despite their different capacities to complete meiotic maturation, suggest that responsiveness to Ras depends on the developmental state of the oocyte and the competence of downstream networks controlling maturation-promoting factor and mitogen-activated protein kinase (MAPK) activation [13,22,23]. However, the upstream regulation of meiotic resumption differs substantially between amphibian and mammalian oocytes. Isolated Xenopus oocytes can undergo maturation in response to progesterone through rapid, transcription-independent signaling [24], whereas mammalian oocytes are maintained in prophase arrest by cyclic guanosine monophosphate (cGMP) supplied from the surrounding follicular somatic cells, which sustains oocyte cyclic adenosine monophosphate (cAMP); gonadotropin-induced remodeling of this somatic-cell network subsequently permits meiotic resumption [25,26,27]. Thus, Xenopus studies demonstrate that Ras-related pathways can influence meiotic entry but do not define the endogenous or isoform-specific functions of Ras within mammalian COCs.

Porcine COCs offer practical and biological advantages for addressing this gap. The approximately 40–44 h porcine IVM interval permits stage-resolved sampling across the germinal vesicle (GV), germinal vesicle breakdown (GVBD)/metaphase I (MI), and metaphase II (MII) stages, while the routine recovery of COCs from abattoir-derived ovaries provides sufficient material for replicated and cell type-specific molecular analyses [28,29]. In addition, pigs share important anatomical and physiological characteristics with humans, including similarities in body and organ size, tissue organization, and several physiological systems, supporting their widespread use as translational large-animal models [30]. Comparative transcriptomic analysis of human, porcine, and mouse oocytes has further revealed both conserved and species-specific programs during meiotic maturation [31]. Together, these characteristics support the use of porcine COCs as a biologically relevant large-animal model for investigating cell type- and stage-dependent molecular regulation during mammalian oocyte maturation.

In addition to Ras family members, Ras activity is regulated by RasGEFs, which promote GDP–GTP exchange, and RasGAPs, which accelerate GTP hydrolysis [32,33,34]. This regulatory balance can shape the amplitude, duration, and subcellular distribution of Ras signaling [35,36]. Among these regulators, SOS1 is a RasGEF, whereas NF1 and RASA1 are RasGAPs [32,33,34]. During the early transition from the GV stage toward meiotic resumption, gonadotropin- and growth factor-derived signals are processed by cumulus cells and activate molecular events leading to GVBD [37,38]. A transient increase in RasGEF-mediated GTP loading could, in principle, facilitate rapid signal transmission during this phase. At the GVBD/MI stage, chromosome condensation and spindle assembly proceed [39], and coordinated RasGEF and RasGAP activity could help constrain Ras output in time and space. During the MI-to-MII transition, polar body extrusion and cortical reorganization occur as the oocyte becomes arrested at MII [40,41,42]; RasGAP-mediated inactivation or spatial redistribution of Ras regulators could contribute to the attenuation or compartmentalization of Ras-dependent signals. These proposed stage-specific roles remain hypothetical because Ras-GTP dynamics and the functional contributions of SOS1, NF1, and RASA1 have not been directly characterized during porcine COC maturation. Accordingly, whether the transcript abundance of these regulators differs according to maturation stage and cell type remains unknown, providing a rationale for examining them separately in cumulus cells and oocytes.

Despite evidence linking Ras-related pathways to meiotic maturation, it remains unknown whether individual Ras isoforms and their regulators exhibit coordinated or divergent patterns between the somatic and germ-cell compartments of porcine COCs. We hypothesized that the distinct physiological and transcriptional states of cumulus cells and oocytes would be reflected in cell type- and stage-dependent patterns of Ras-related transcript abundance during IVM. Specifically, cumulus cells were expected to exhibit broader maturation-associated expression changes related to cumulus expansion and remodeling of intercellular communication, whereas oocytes were expected to display more molecule-specific temporal patterns reflecting meiotic progression and reliance on stored maternal mRNAs. To test this hypothesis, cumulus cells and oocytes were analyzed separately at 0, 22, and 44 h of IVM, which were selected to capture the immature GV phase, the GVBD/MI transition, and the MII phase, respectively [43]. This design was intended to prevent cell type-specific or opposing patterns from being obscured in whole-COC measurements and to distinguish early, intermediate, and late maturation-associated changes. Transcript abundance was examined for *H-Ras*, *K-Ras*, *N-Ras*, *R-Ras*, *NF1*, *RASA1*, and *SOS1*. *Luteinizing hormone receptor* (*LHR*), *follicle-stimulating hormone receptor* (*FSHR*), *epidermal growth factor receptor* (*EGFR*), and *CX43* transcripts were also analyzed to contextualize maturation-associated changes in receptor-related signaling and intercellular communication. This study therefore aimed to determine whether Ras-related transcript abundance exhibits uniform or cell type-, molecule-, and stage-dependent transcript patterns during porcine IVM.

## 2. Materials and Methods

### 2.1. IVM

The source of the porcine ovaries was discarded by-products from a commercial slaughterhouse in Pocheon, Republic of Korea, and they were collected for research purposes with permission from the slaughterhouse and transported to the laboratory within 2 h in 0.9% (*w*/*v*) saline maintained at 38 °C. Upon arrival, the ovaries were washed at least three times with 0.85% (*w*/*v*) saline. Porcine COCs were aspirated from antral follicles measuring 3–6 mm in diameter using an 18-gauge needle connected to a disposable syringe. Using a stereomicroscope (SZX16, Olympus, Tokyo, Japan), the aspirated COCs were transferred into pre-incubated phosphate-buffered saline containing polyvinyl alcohol (PBS–PVA; catalog no. 21300025; Thermo Fisher Scientific, Waltham, MA, USA) using a mouse pipette. Only COCs with a homogeneous ooplasm and more than three compact layers of cumulus cells were selected for IVM.

Porcine follicular fluid (pFF) was collected from aspirated follicular contents, and the upper fluid fraction was transferred into 50 mL conical tubes. The collected follicular fluid was centrifuged at 850× *g* for 45 min at 4 °C (1248R, Labogene, Lynge, Denmark), and the supernatant was carefully recovered. This centrifugation procedure was repeated three times in total to remove cells and particulate debris. The final supernatant was filtered twice through a 0.45 μm syringe filter and stored at −20 °C until use.

Selected COCs were cultured in 650 µL of tissue culture medium-199 (TCM-199; catalog no. 12340-030; Invitrogen, Carlsbad, CA, USA) supplemented with 0.5 µg/mL FSH (catalog no. F2293; Sigma, St. Louis, MO, USA), 10 ng/mL EGF (catalog no. E-4127; Sigma), 10 IU/mL human chorionic gonadotropin (hCG; Intervet, Boxmeer, The Netherlands), and 10% (*v*/*v*) pFF at 38.5 °C in an atmosphere of 5.0% CO_2_ for 22 h, following a laboratory protocol previously used in our laboratory for subsequent porcine in vitro fertilization and embryo development [44]. This two-stage IVM protocol was used to provide gonadotropin stimulation during the initial phase of maturation and to allow continued maturation without prolonged exposure to FSH and hCG during the subsequent phase.

After the first 22 h of culture, intact COCs were gently transferred using a 100 µL micropipette and sequentially washed in three drops of fresh, pre-equilibrated second-stage medium to minimize the carryover of FSH and hCG from the first-stage medium. The washed COCs were then transferred into 650 µL of fresh TCM-199 supplemented with 10 ng/mL EGF and 10% (*v*/*v*) pFF, but without FSH or hCG. To preserve the integrity of the cumulus cell layers and cell–cell contacts, the COCs were handled by gentle aspiration and dispensing, while vigorous or repeated pipetting was avoided. The washing and transfer procedures were performed promptly to minimize exposure to conditions outside the incubator. The COCs were subsequently cultured for an additional 22 h under the same temperature and atmospheric conditions.

For subsequent analyses, COCs were collected at 0, 22, and 44 h after the onset of IVM, washed in PBS–PVA, and immediately processed according to the requirements of each downstream analysis. In a separate set of experiments, morphological changes in COCs during IVM were monitored under the same culture conditions using an EVOS M7000 system (Thermo Fisher Scientific), with images acquired at 3 h intervals.

### 2.2. Collection of Cumulus Cells and Oocytes

For gene expression analysis, each group of COCs was treated with 0.1% (*w*/*v*) hyaluronidase (catalog no. H3506; Sigma) in PBS–PVA for 10 min at 38.5 °C, followed by additional pipetting using a mouse pipette with a diameter adjusted to the approximate size of the oocytes to ensure complete separation of cumulus cells from the oocytes. The enzymatic reaction was terminated by immediately transferring the separated cumulus cells and oocytes into enzyme-free PBS–PVA, followed by three washes with fresh PBS–PVA to remove residual hyaluronidase. The use of 0.1% hyaluronidase followed by repeated washing has previously been applied to the separation of porcine oocytes and cumulus cells for subsequent cell type-specific gene expression analyses [45,46]. The separated cumulus cells and oocytes were stored at −80 °C until further use for gene expression analysis.

### 2.3. Real-Time PCR

For gene expression analysis, 60 COCs were collected at each maturation time point for each biological replicate and separated into cumulus cells and oocytes. Total RNA was extracted separately from cumulus cell and oocyte samples collected at 0, 22, and 44 h of IVM using an easy-spin™ Total RNA Extraction Kit (catalog no. 17221; iNtRON Biotechnology, Seoul, Republic of Korea). RNA concentration was measured using an EzDrop 1000C spectrophotometer (Blue-Ray Biotech, New Taipei City, Taiwan), and cDNA was synthesized using the SuPrimeScript cDNA Synthesis Kit (catalog no. SRK-1000; GeNeTBio, Daejeon, Republic of Korea), according to the manufacturer’s instructions.

Real-time polymerase chain reaction (RT-PCR) was performed using TOPreal™ SYBR Green qPCR PreMIX (catalog no. RT500M; Enzynomics, Daejeon, Republic of Korea) on a QuantStudio 3 Real-Time PCR System (Applied Biosystems, Waltham, MA, USA) following the manufacturer’s instructions. The gene expression levels of gonadotropin and growth factor receptors (*LHR*, *FSHR*, and *EGFR*), Ras subfamily members (*R-Ras*, *K-Ras*, *H-Ras*, and *N-Ras*), RasGAP-associated genes (*NF1* and *RASA1*), RasGEF-associated gene (*SOS1*), and a cell–cell junction-related gene (*CX43*) were analyzed. The mRNA expression levels were normalized to *β*-actin, and relative expression levels were calculated using the comparative cycle threshold (2^−ΔΔCt^) method. The primer sequences used in this study are listed in Table 1. Because oocyte hormone receptor-related transcripts were not consistently detected by RT-PCR, semi-quantitative PCR and agarose gel electrophoresis were additionally performed for *LHR*, *FSHR*, and *EGFR*, as described in the following section.

### 2.4. Quantitative Reverse Transcription PCR

PCR amplification and band intensity analysis were performed to further evaluate oocyte hormone receptor-related transcripts. Oocyte cDNA samples synthesized as described above were amplified using AccuPower^®^ PCR PreMix & Master Mix (catalog no. K-2016; Bioneer, Seoul, Republic of Korea) and a Veriti 96-well Thermal Cycler (Applied Biosystems), following the manufacturer’s instructions. The PCR products were separated by electrophoresis on a 1.5% agarose gel containing RedSafe Nucleic Acid Staining Solution (catalog no. 21141; iNtRON Biotechnology) at 90 V for 25 min and photographed under UV illumination. *LHR* band intensities were quantified using ImageJ software version 1.54 (National Institutes of Health, NIH, Bethesda, MD, USA), normalized to *β*-actin, and expressed relative to the 0 h group. *FSHR* and *EGFR* amplification products were not consistently detected at the expected sizes under the assay conditions and were therefore not subjected to densitometric or statistical analysis; these results were designated as not detected (ND). Representative gel images are presented in Supplementary Figure S1. The primer sequences used in this study are listed in Table 1.

### 2.5. Statistical Analysis

Each experiment was performed at least three times. All data were analyzed using GraphPad Prism version 5.03 (GraphPad Software Inc., San Diego, CA, USA). Data are reported as the mean ± standard error of the mean (SEM). Differences among the 0, 22, and 44 h groups were analyzed using one-way analysis of variance (ANOVA), followed by Tukey’s multiple-comparisons test. Statistical significance is indicated by * *p* < 0.05, ** *p* < 0.01.

## 3. Results

### 3.1. Morphological and Molecular Changes During Maturation in Porcine COCs

**Figure 1:**
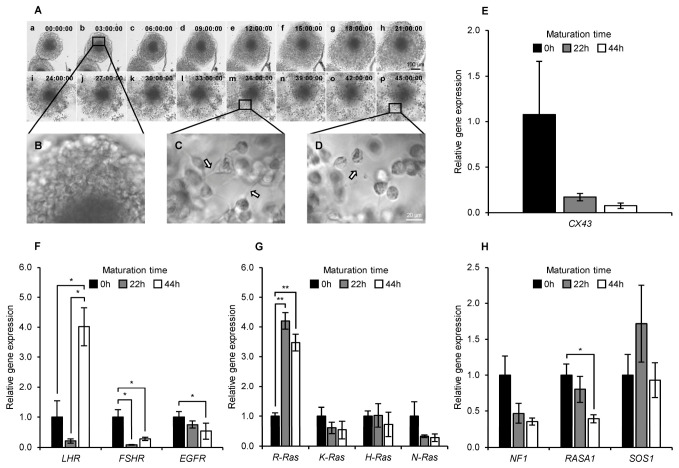
Morphological changes and gene expression patterns in porcine cumulus-oocyte complexes (COCs) during in vitro maturation (IVM). Porcine COCs were cultured under IVM conditions supplemented with follicle-stimulating hormone (FSH), human chorionic gonadotropin (hCG), and epidermal growth factor (EGF) and continuously observed from 0 to 45 h using a time-lapse imaging system (**A**). Representative bright-field images were captured at 3 h intervals (a–p), showing progressive cumulus cell expansion during maturation. As IVM proceeded, dynamic morphological changes associated with cumulus cell expansion and cell–cell contacts were observed within the cumulus mass. Higher-magnification images (**B**–**D**) highlight regions within the cumulus mass where altered cell–cell contact structures became apparent during expansion (white arrows). Scale bars indicate 100 µm in (**A**) and 20 µm in (**D**). Relative mRNA expression of the gap junction-related gene *CX43* (*Connexin 43*) is shown in (**E**). Relative mRNA expression levels of hormone receptor genes, including *luteinizing hormone receptor* (*LHR*), *follicle-stimulating hormone receptor* (*FSHR*), and *epidermal growth factor receptor* (*EGFR*), are shown in (**F**). Relative mRNA expression levels of Ras subfamily genes, including *related Ras viral oncogene* (*R-Ras*), *Kirsten rat sarcoma virus* (*K-Ras*), *Harvey rat sarcoma virus* (*H-Ras*), and *neuroblastoma RAS viral oncogene homolog* (*N-Ras*), are shown in (**G**). Relative mRNA expression levels of RasGAP and RasGEF genes, including *Neurofibromin 1* (*NF1*), *RAS p21 protein activator 1* (*RASA1*), and *Son of sevenless homolog 1* (*SOS1*), are shown in (**H**). The gene expression analyses shown in (**E**–**H**) were performed using three independent biological replicates (*n* = 3). Data are presented as the mean ± standard error of the mean (SEM). Differences among the 0, 22, and 44 h groups were analyzed using one-way analysis of variance (ANOVA) followed by Tukey’s multiple-comparisons test. Brackets indicate the compared groups, and asterisks indicate significant differences (* *p* < 0.05, ** *p* < 0.01).

Morphological changes in porcine COCs during maturation are shown in Figure 1A. Progressive cumulus cell expansion was observed from 0 to 45 h of IVM (Figure 1A). Higher-magnification images (Figure 1B–D) showed altered cell–cell contact structures within the cumulus mass as maturation progressed.

The expression pattern of the gap junction gene *CX43* during porcine cumulus cell maturation is shown in Figure 1E. *CX43* mRNA expression did not differ significantly among the maturation times.

The expression patterns of hormone receptor genes during the maturation of porcine cumulus cells are shown in Figure 1F. *LHR* mRNA expression was significantly higher at 44 h than at both 0 and 22 h (*p* < 0.05). *FSHR* mRNA expression was significantly lower at 22 and 44 h than at 0 h (*p* < 0.05), with no significant difference between 22 and 44 h. *EGFR* mRNA expression was significantly lower at 44 h than at 0 h (*p* < 0.05).

The expression patterns of Ras subfamily genes during maturation in porcine cumulus cells are shown in Figure 1G. *R-Ras* mRNA expression was significantly higher at both 22 and 44 h than at 0 h (*p* < 0.01). *K-Ras*, *H-Ras*, and *N-Ras* mRNA expression did not differ significantly among the maturation times.

The expression patterns of RasGAP and RasGEF during the maturation of porcine cumulus cells are shown in Figure 1H. *NF1* mRNA expression did not differ significantly among the maturation times. *RASA1* mRNA expression was significantly decreased at 44 h compared to that at 0 h (*p* < 0.05). *SOS1* mRNA expression did not differ significantly among the maturation times.

### 3.2. Changes in Hormone Receptor and Ras-Related Gene During Maturation in Porcine Oocytes

**Figure 2:**
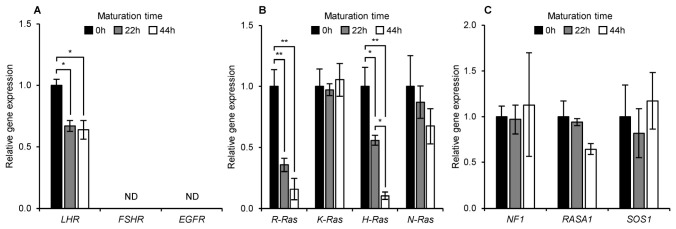
Changes in hormone receptor and Ras-related gene expression during maturation in porcine oocytes. Relative mRNA expression of *LHR* and the detection patterns of *FSHR* and *EGFR* are shown in (**A**). *FSHR* and *EGFR* transcripts were not consistently detected under the assay conditions and are indicated as ND; these targets were not included in quantitative or statistical analyses. Relative mRNA expression levels of Ras subfamily genes, including *R-Ras*, *K-Ras*, *H-Ras*, and *N-Ras*, are shown in (**B**). Relative mRNA expression levels of RasGAP and RasGEF genes, including *NF1*, *RASA1*, and *SOS1*, are shown in (**C**). The numbers of independent biological replicates were as follows: *LHR*, *n* = 4; *FSHR* and *EGFR* were each examined in three independent biological replicates; *R-Ras*, *n* = 3; *K-Ras*, *n* = 5; *H-Ras*, *n* = 3; *N-Ras*, *n* = 4; and *NF1*, *RASA1*, and *SOS1*, *n* = 3 each. Data are presented as the mean ± SEM. Differences among the 0, 22, and 44 h groups were analyzed using one-way ANOVA followed by Tukey’s multiple-comparisons test. Brackets indicate the compared groups, and asterisks indicate significant differences (* *p* < 0.05, ** *p* < 0.01). ND, not detected under the assay conditions.

The expression patterns of hormone receptor-related genes during porcine oocyte maturation are shown in Figure 2A. *LHR* mRNA expression was significantly lower at 22 and 44 h than at 0 h (*p* < 0.05). *FSHR* and *EGFR* transcripts were not consistently detected under the assay conditions and are therefore indicated as not detected (ND).

The expression patterns of Ras subfamily genes in porcine oocytes during IVM are shown in Figure 2B. *R-Ras* mRNA expression was significantly lower at both 22 and 44 h than at 0 h (*p* < 0.01), with no significant difference between 22 and 44 h. *K-Ras* mRNA expression did not differ significantly among the maturation times. *H-Ras* mRNA expression was significantly lower at 22 h than at 0 h (*p* < 0.05) and was further decreased at 44 h compared with both 0 h (*p* < 0.01) and 22 h (*p* < 0.05). *N-Ras* mRNA expression did not differ significantly among the maturation times.

The expression patterns of RasGAP and RasGEF in porcine oocytes during IVM are shown in Figure 2C. *NF1*, *RASA1*, and *SOS1* mRNA expression levels did not differ significantly among maturation time points.

## 4. Discussion

In this study, we examined morphological changes during porcine IVM and analyzed the transcript abundance of hormone receptor-related factors, *CX43*, and Ras-related components in cumulus cells and oocytes. As cumulus cells and oocytes undergo distinct functional transitions during maturation, the results are discussed with particular attention to cell type- and stage-dependent differences.

Morphological observations using time-lapse imaging revealed progressive cumulus expansion during IVM. Higher-magnification images showed apparent changes in cell–cell contact morphology within the cumulus mass as maturation progressed. Cumulus expansion is an important feature of COC maturation and reflects functional changes in cumulus cells, including extracellular matrix remodeling, loosening of intercellular contacts, and altered responsiveness to maturation-associated signaling [47,48,49]. Alongside progressive cumulus expansion, selected Ras-related transcripts also changed during IVM, including increased *R-Ras* transcript abundance and lower *RASA1* transcript abundance at later maturation stages in cumulus cells. These Ras-related transcript changes occurred alongside the progressive structural reorganization of the cumulus mass during maturation. However, because the morphological observations and molecular measurements were obtained from separate experimental samples, a direct sample-level statistical correlation or causal relationship could not be established in the present study. Future studies should quantify cumulus expansion and Ras-related transcript changes in matched COCs and use targeted inhibition or manipulation of individual Ras-related factors to determine their relationships with extracellular matrix remodeling and cumulus expansion.

The *CX43* results provide additional context for maturation-associated changes in intercellular communication. *CX43* transcript abundance did not differ significantly among the maturation time points. Gap junction-mediated communication between cumulus cells and between cumulus cells and oocytes is essential during early maturation, as it facilitates the exchange of small molecules, metabolites, and signaling intermediates. However, this communication is altered by meiotic resumption and cumulus expansion [49]. Previous studies have shown that gonadotropin-induced MAPK activation promotes *CX43* phosphorylation and reduces gap junctional communication in mammalian ovarian follicles [27], whereas gonadotropin-dependent redistribution of *CX43* accompanies gap-junction remodeling in porcine COCs [9]. Because Ras is positioned upstream of the rapidly accelerated fibrosarcoma (Raf)–MAPK/ERK kinase (MEK)–ERK cascade, these findings support a biologically plausible regulatory direction from Ras/MAPK signaling toward *CX43* functional remodeling rather than establishing *CX43* as an upstream regulator of Ras. However, the present study measured *CX43* transcript abundance and Ras-related transcript abundance, but not GTP-bound Ras, ERK activation, *CX43* phosphorylation, or gap-junction permeability; therefore, the present data cannot establish a direct upstream–downstream relationship.

Hormone receptor-related transcripts in cumulus cells exhibited nonuniform temporal patterns during IVM, characterized by late-stage elevation of *LHR* and lower *FSHR* and *EGFR* transcript abundance at one or more later maturation stages. FSH and EGF facilitate cumulus expansion and oocyte maturation during the early phase of IVM, whereas subsequent stages are accompanied by differentiation-associated changes in cumulus cell function [50,51,52]. These patterns may therefore reflect stage-dependent changes in receptor-related responsiveness and cumulus cell differentiation rather than uniform maintenance of receptor expression throughout maturation. However, because receptor activation and downstream signaling were not directly measured, the functional consequences of these transcript changes remain unclear.

Ras-related transcripts in cumulus cells exhibited molecule-specific rather than uniform patterns during IVM. R-Ras transcript abundance increased at 22 and 44 h, whereas *RASA1* transcript abundance was lower at 44 h. In contrast, *H-Ras*, *K-Ras*, *N-Ras*, *NF1*, and *SOS1* transcript abundance did not differ significantly among the maturation time points. These findings indicate that only selected components of the Ras-related regulatory network exhibited temporal transcript changes during cumulus cell maturation. However, because Ras activity is determined by the balance between its GTP-bound active and GDP-bound inactive states [5], the observed transcript patterns cannot be interpreted as evidence of altered Ras activation or downstream pathway activity. Corresponding protein-level analyses, direct measurement of GTP-bound Ras, and assessment of downstream signaling effectors are required to determine the functional significance of these transcript patterns.

The transcript patterns in oocytes differed from those in cumulus cells. The most prominent contrast was the opposing trajectory of *R-Ras* transcript abundance between the two cellular compartments: *R-Ras* transcript abundance increased in cumulus cells but decreased in oocytes during IVM. The other Ras isoforms also did not exhibit uniform patterns across oocytes and cumulus cells. This divergence is consistent with cell type- and isoform-specific Ras-related transcript patterns during IVM rather than a common regulatory program operating uniformly throughout the COC [53].

When cumulus cells and oocytes were considered together, Ras-related transcripts exhibited distinct patterns between the two cell types during porcine IVM. This divergence may reflect fundamental differences in their biological functions, transcriptional states, and modes of signal reception. Cumulus cells remain transcriptionally responsive and are directly exposed to the extracellular IVM environment, including gonadotropin and growth-factor cues during the initial culture phase, while undergoing extracellular-matrix formation, cumulus expansion, and remodeling of intercellular junctions. In contrast, fully grown oocytes are largely transcriptionally quiescent and depend on stored maternal mRNAs during meiotic maturation. Thus, changes in oocyte transcript abundance may reflect molecule-specific differences in the stability or utilization of stored maternal transcripts, whereas changes in cumulus cells may involve active transcriptional responses to the IVM environment. These biological differences may contribute to the cell type- and molecule-specific transcript patterns observed during IVM, although the underlying mechanisms remain to be determined.

A major limitation of this study is that the molecular analyses were restricted to transcript abundance and did not assess corresponding protein abundance, subcellular distribution, GTP-bound Ras, or downstream pathway activity. Therefore, the observed transcript patterns cannot be interpreted as direct evidence of changes in protein abundance or Ras activation. Future studies should include validated protein-level analyses with appropriate specificity controls, direct Ras-GTP activity assays, and assessment of downstream signaling effectors. In addition, because a hormone- and growth factor-free control group was not included, the present experimental design cannot distinguish changes associated with spontaneous maturation under in vitro conditions from those specifically induced by FSH, hCG, or EGF. Furthermore, no knockdown, overexpression, or other functional perturbation of individual Ras-related factors was performed; therefore, their causal roles in cumulus expansion, meiotic progression, or oocyte competence could not be established. Future studies incorporating appropriate control groups and targeted genetic or pharmacological manipulation will be required to determine the functional significance of the observed transcript patterns.

## 5. Conclusions

This study showed that porcine IVM is accompanied by cumulus expansion and cell type- and stage-dependent patterns in the transcript abundance of selected hormone receptor-related factors, *CX43*, Ras family members, and their RasGAP and RasGEF regulators in COCs. In cumulus cells, IVM was associated with selected changes in hormone receptor-related and Ras-related transcripts, whereas *CX43* transcript abundance did not differ significantly among the maturation time points. In oocytes, Ras-related transcript abundance exhibited molecule-specific temporal patterns, with R-Ras showing an opposite trajectory to that observed in cumulus cells. These findings provide a foundation for understanding cell type- and stage-specific Ras-related transcript patterns in cumulus cells and oocytes during porcine IVM and highlight the need for validated protein-level analyses with appropriate specificity controls, direct functional analyses, and measurements of Ras activity.

## Figures and Tables

**Table 1:** Primer conditions used in this study.

Gene	Sequence (5′–3′)	Annealing Temp. (°C)	Cycle	Product Size (bp)	Accession No.	PCR Type
*CX43*	F: TCATCTTCATGCTGGTCGTATC	60	40	99	NM_001244212.1	RT-PCR
	R: TTCCCTTCACACGATCCTTAAC					
*LHR*	F: GGAGAATGCACACCTGAAGA	60	40	94	JN120794.1	RT-PCR
	R: GGCCTGTAGTTTAGTGGAAGAA					
*LHR*	F: CTGTCCTCTTTGTTCTCCTGAC	60	34	191	NM_214449.1	qPCR
	R: AGCAACACTACACCCATTCC					
*FSHR*	F: CTGCCTGCCCATGGATATT	60	40	104	NM_214386.3	RT-PCR
	R: GAGTATAGCAGCCACAGATGAC					
*FSHR*	F: GATCCTGATCACCAGCCAATAC	60	34	217	NM_214386	qPCR
	R: GACTGAGAGCTCACTAGCAAAG					
*EGFR*	F: CCCAGCACCCGAATATGTAA	60	40	87	NM_214007.1	RT-PCR
	R: CTGAGAGGCTGATTGTGGTAG					
*EGFR*	F: CCTTGGGAACTTGGAGATCACCTAC	60	34	344	NM_214007	qPCR
	R: TGTTGCTTAGAAAGTCGCTGTTGAC					
*R-Ras*	F: CCTGCTGGTGTTTGCCATTA	60	40	94	XM_003355998.3	RT-PCR
	R: GAAGTCATCTCGGTCCTTGACT					
*K-Ras*	F: GATGGAGAAACCTGTCTCTTGG	60	40	80	XM_005653151	RT-PCR
	R: CTCATGTACTGGTCCCTCATTG					
*H-Ras*	F: CCCTGACCATCCAGCTTAT	60	40	89	XM_021082554.1	RT-PCR
	R: GTCAATGACCACTTGCTTCC					
*N-Ras*	F: AACCAGACAGGGTGTTGAAG	60	40	103	NM_001044537.1	RT-PCR
	R: ACAACCTTGAGTCCCATCATC					
*NF1*	F: GCAGTTCAGACCCTAGTTTAC	60	40	123	XM_021067460.1	RT-PCR
	R: TGTTGGCTGGGATACATAACC					
*RASA1*	F: TCCAGAACAAGCAGAGGATTG	60	40	97	XM_021084515.1	RT-PCR
	R: GACCTGACGCAGACGTTTAT					
*SOS1*	F: TCCTCCTGCTTCTGGTGCTTCTAG	60	40	97	XM_021087589.1	RT-PCR
	R: AAAGACGGTATCGCTGCTTGAGTG					
*β* *-actin*	F: TCTGGCACCACACCTTCTA	60	40	102	XM_021086047.1	RT-PCR
	R: TCTTCTCACGGTTGGCTTTG					
*β* *-actin*	F: GGACTTCGAGCAGGAGATGG	60	32	233	XM_003124280	qPCR
	R: GCACCGTGTTGGCGTAGAGG					

Abbreviations: RT-PCR, real-time polymerase chain reaction; qPCR, quantitative reverse transcription polymerase chain reaction.

## Data Availability

All data generated or analyzed during this study are included in this published article.

## Supplementary Materials

The following supporting information can be downloaded at: https://www.mdpi.com/article/10.3390/ani16142125/s1, Figure S1: Representative agarose gel electrophoresis of quantitative PCR products for hormone receptor-related genes in porcine oocytes during in vitro maturation (IVM).
