# Supplementary material for: Changes in Ras and Ras-Associated GTPases During Maturation of Porcine Cumulus Cells and Oocytes
Source: Animals (Basel). 2026 Jul 9;16(14):2125. doi: 10.3390/ani16142125 (PMC13405820; doi:10.3390/ani16142125)
Supplement: Supplementary file 1 [file animals-16-02125-s001.zip › animals-4374525-supplementary.pdf]

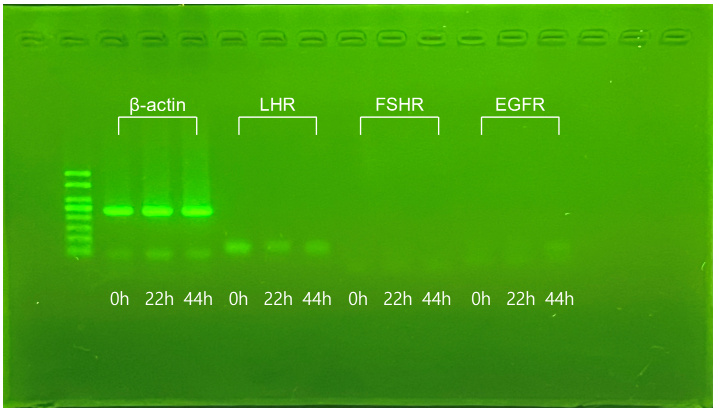

Supplementary Figure S1. Representative agarose gel electrophoresis of quantitative PCR products for hormone receptor-related genes in porcine oocytes during in vitro maturation (IVM). Quantitative PCR was performed for *LHR*, *FSHR*, and *EGFR* using oocyte samples collected at 0, 22, and 44 h of IVM. *β-actin* was used as the internal control. PCR products were separated on a 1.5% agarose gel.
